# Genome of *Ganoderma* Species Provides Insights Into the Evolution, Conifers Substrate Utilization, and Terpene Synthesis for *Ganoderma tsugae*

**DOI:** 10.3389/fmicb.2021.724451

**Published:** 2021-09-16

**Authors:** Nan Jiang, Shuang Hu, Bing Peng, Zhenhao Li, Xiaohui Yuan, Shijun Xiao, Yongping Fu

**Affiliations:** ^1^International Cooperation Research Center of China for New Germplasm Breeding of Edible Mushrooms, Jilin Agricultural University, Changchun, China; ^2^Shouxiangu Botanical Drug Institute Co., Ltd., Jinhua, China; ^3^Jiaxing Key Laboratory for New Germplasm Breeding of Economic Mycology, Jiaxing, China

**Keywords:** *Ganoderma*, whole-genome sequencing, PacBio long reads, comparative genomics, plant-pathogen interaction, terpene synthases

## Abstract

*Ganoderma tsugae* is an endemic medicinal mushroom in Northeast China, providing important source of pharmaceutical product. Comparing with other *Ganoderma* species, wild *G. tsugae* can utilize coniferous wood. However, functional genes related to medicinal component synthesis and the genetic mechanism of conifer substrate utilization is still obscure. Here, we assembled a high-quality *G. tsugae* genome with 18 contigs and 98.5% BUSCO genes and performed the comparative genomics with other *Ganoderma* species. *G. tsugae* diverged from their common ancestor of *G. lingzhi* and *G. sinense* about 21 million years ago. Genes in *G. tsugae*-specific and *G. tsugae*-expanded gene families, such as *salh*, *phea*, *cyp53a1*, and *cyp102a*, and positively selected genes, such as *glpk* and *amie*, were functionally enriched in plant-pathogen interaction, benzoate degradation, and fanconi anemia pathway. Those functional genes might contribute to conifer substrate utilization of *G. tsugae*. Meanwhile, gene families in the terpene synthesis were identified and genome-wide SNP variants were detected in population. Finally, the study provided valuable genomic resources and offered useful hints for the functional gene mapping and investigation of key gene contributing to conifer cultivation substrate utilization and medicinal component biosynthesis.

## Introduction

*Ganoderma tsugae* is a precious cultivated *Ganoderma* species in Northeast China, which has become an important source of pharmaceutical product (Hung et al., 2004; Wu et al., 2004). Compared with other cultivated *Ganoderma* species, such as *G. lingzhi* and *G. sinense*, wild *G. tsugae* mainly distribute in the Changbai Mountain and grow on coniferous wood, such as *Larix gmelinii*, *Abies nephrolepis*, *Abies fabri*, etc. Moreover, the culture temperature of *G. tsugae* is lower than that of *G. lingzhi* (Adaskaveg and Gilbertson, 1986). Previous studies mainly focused on the isolation and pharmaceutical functions of secondary metabolites in *G. tsugae* (Kıvrak, 2015; Ma et al., 2020). Identifying key genes for conifer substrate utilization and medicinal component synthesis would facilitate our understanding for the mechanism of habitat environmental adaptation and molecular improvement of *G. tsugae*; however, genetic basis underlying those important biological processes is still unclear. Besides, the molecular markers and genetic diversity analysis of *G. tsugae* germplasm resources are rarely reported before.

The edible and medicinal fungi contain a large number of active metabolites, such as terpenes, alkaloids, and polyketides, which have a wide range of pharmaceutical applications (Song et al., 2016). In recent years, many studies have been carried out on the pharmacological activities of *G. tsugae* and showed that it has many pharmacological properties, such as antioxidant (Mau et al., 2002), anti-inflammation (Ko et al., 2008), anti-tumor (Yu et al., 2012; Kuo et al., 2013; Huang et al., 2019), lipid-lowering (Tseng et al., 2018), immunity enhancement (Jinn et al., 2006), etc. The rapid development of sequencing technology and genomic data mining enable scientists to elaborately investigate functional genes of important bioactive compounds in fungi. Many medicinal fungi such as *G. lingzhi* (Chen et al., 2012; Liu et al., 2012; Huang et al., 2013), *G. sinense* (Zhu et al., 2015), *Antrodia cinnamomea* (Lu et al., 2014), *Hericium erinaceus* (Gong et al., 2020), and *Gloeostereum incarnatum* (Wang et al., 2019) have been reported, and gene clusters related to their pharmacological properties have been speculated. In *G. lingzhi*, 15 genes that may be involved in triterpene biosynthesis were identified based on the structural similarity of steroids and triterpenes (Chen et al., 2012). Based on genome sequence of *Flammulina velutipes*, 15 genes related to the synthesis of terpene backbone, 19 genes related to steroid synthesis, and three genes related to terpene and triterpene synthesis were identified (Park et al., 2014). However, metabolic pathways and functional genes related with important bioactive compounds have not yet been identified and characterized for *G. tsugae*. Although a genome of *G. tsugae* s90 strain (GCA_003057275.1) has been released, the genome is constructed using traditional short reads sequencing technique, leading to a fragmented assembly, with 6,742 contigs and a N50 of 11.7 kb, and hindering the investigation of functional gene clusters for important bioactive compounds.

Here, we provide a high-quality genome assembly of *G. tsugae* and performed genomic comparison with those of broadleaf-tree parasitic *G. lingzhi*, *G. sinense*, and *G. boninense*. Populations of *G. tsugae* and *G. lingzhi* were sequenced to identified genome-wide molecular markers. The aims of this study are as follows: (1) generate a high-quality reference genome for *G. tsugae* to lay a solid foundation for further functional genomic studies; (2) provide hints for the mechanism of conifer substrate utilization by comparative genomic studies between *G. tsugae* and three broadleaf-tree parasitic *Ganoderma* species; (3) identify putative functional genes involved in the pharmaceutical compound synthesis in the genome; and (4) develop genome-wide molecular markers in a high-throughput manner from population sequencing for *G. tsugae*.

## Materials and Methods

### Strains and DNA Extraction

A protoplast-derived monokaryon (CCMJ4178) from the wild dikaryotic *G. tsugae* strain CCMJ2475, which was collected from the Changbai Mountain in northeastern China, was isolated following our previous study (Dai et al., 2017). Mycelia were cultured at 25°C with 150 rpm/min for 10 days and then incubated for 3 h at 28°C in lywallzyme lysing enzyme. The obtained monokaryon was then used for *de novo* genome sequencing. Twenty-two *G. tsugae* and 18 *G. lingzhi* strains were used in the whole-genome resequencing (Supplementary Table 1). All strains used in this study were maintained at the Engineering Research Center of Chinese Ministry of Education for Edible and Medicinal Fungi at the Jilin Agricultural University. Their mycelia were quickly frozen in liquid nitrogen and then used for DNA extraction using the NuClean Plant Genomic DNA Kit (CWBIO, Beijing, China) according to the instructions.

### *De novo* Genome Sequencing, Assembly, and Annotation

The genome of monokaryon *G. tsugae* strain (CCMJ4178) was sequenced using the PacBio Sequel long-read sequencing and Illumina NovaSeq platforms with the 20-kb and 350-bp insert size, respectively. *De novo* assembly was conducted by SMARTdenovo^1^ with PacBio long reads. Then, two rounds of sequence polishing with Illumina short reads were performed using Pilon software (Walker et al., 2014). The integrity of the final assembly was assessed by Benchmarking Universal Single-Copy Orthologs (BUSCO) (Simão et al., 2015; Waterhouse et al., 2018). Finally, this assembly was submitted to GenBank under a BioProject PRJNA733861.

To annotate repetitive elements in the assembled genome, *de novo* and homology-based approaches using RepeatModeler^2^ and RepeatMasker^3^ were performed, and prediction results were combined for the final repetitive element annotation. Tandem Repeats Finder^4^ and MIcroSAtellite^5^ were used to identify tandem repeats and simple sequence repeats (SSR), respectively. Mononucleotide repeats more than 10 times, dinucleotide repeats more than six times, and tri-, tetra-, penta-, and hexa-nucleotide repeats more than five times were defined as SSRs. Primers were designed using Primer 3.

To systematically annotate genes in the assembled genome, we integrated gene model predictions from *de novo*, homolog, and RNA-seq based methods. Augustus (Stanke et al., 2006), SNAP (Korf, 2004), GeneScan (Burge and Karlin, 1997), and GlimmHmm (Majoros et al., 2004) were used for *de novo* gene predictions. For homolog gene-based prediction, proteomes of *Agaricus bisporus* (Morin et al., 2012), *Coprinopsis cinerea* (Stajich et al., 2010), *Pleurotus ostreatus* (Qu et al., 2016), and *Schizophyllum commune* (Ohm et al., 2010) were aligned using tBLASTn and Genewise for homologous protein mapping (Birney et al., 2004). At last, transcriptome sequence reads were aligned to the genome using TopHat package (Trapnell et al., 2009), and the gene structure was predicted using Cufflinks (Ghosh and Chan, 2016). Finally, all gene models were integrated and redundancy was eliminated by MAKER (Cantarel et al., 2008). To functionally annotate *G. tsugae* genes, we searched the predicted genes against NCBI Non-redundant database (Nr), Nucleotide Sequence Database (Nt), Eukaryotic Clusters of Orthologous Groups (KOG) (Tatusov et al., 2000), SwissProt (Boutet et al., 2016), Pfam, Gene Ontology (GO), and Kyoto Encyclopedia of Genes and Genomes (KEGG)^6^ databases. Meanwhile, tRNAscan-SE (Lowe and Eddy, 1997), RNAmmer (Lagesen et al., 2007), and Rfam (Gardner et al., 2009) were used to predict non-coding genes.

### Carbohydrate-Active Enzyme Family and Secondary Metabolite Annotations

We used dbCAN2 meta server (Zhang et al., 2018) to identify the carbohydrate-active enzymes (CAZymes) in genomes of *G. tsugae*, *G. lingzhi*, *G. sinense*, and *G. boinense*^7^ by mapping the protein sequences to the CAZy database (Cantarel et al., 2009). The gene clusters associated with secondary metabolic biosynthesis of these four *Ganoderma* species were searched using antiSMASH 6 beta.^8^

### Gene Family Identification

Whole-genome protein-coding genes of *G. tsugae*, three previously reported *Ganoderma* species (*G. lingzhi*, *G. sinense*, *G. boninense*), three other Polyporaceae species [*Trametes coccinea* (Couturier et al., 2015), *T. pubescens*,^9^ *Dichomitus aqualens* (Floudas et al., 2012)], and four fossil record fungi species [*Coprinopsis cinerea* (Stajich et al., 2010), *Coniophora puteana* (Floudas et al., 2012), *Laccaria bicolor* (Martin et al., 2008), *Serpula lacrymans* (Eastwood et al., 2011)] were used for gene family identification and analysis. Only proteins from the longest transcript were used for genes. Firstly, proteins from *G. tsugae* and the closely related species were all-to-all blasted by BLASTP with an *e*-value threshold of 1*e*-5. OrthoMCL v1.2 (Li et al., 2003) was used to cluster gene families based on the above blast result. Single-copy orthologs are genes with one and only one copy in all species. The conservation synteny of *G. tsugae*, *G. lingzhi*, *G. sinense*, and *G. boninense* was analyzed using MCscan with default parameters.

### Phylogenetic Analysis for *G. tsugae* and Other Species

Using single-copy orthologs, we could probe the phylogenetic relationships for *G. tsugae* and other species. To this end, protein sequences of single-copy genes were aligned using CLUSTALX2.0. Based on the protein multisequence alignment, coding DNA sequences (CDS) alignment were generated and concatenated. Gblocks (Castresana, 2000) was then applied to remove low-quality alignments in highly variable regions. The phylogenic relationships of those species were constructed using PhyML3.0 by maximum likelihood method with 100 bootstraps using the JTT + G + F model. The recalibration for the divergence of 70.0∼129.4 MYA between *S. lacrymans* and *C. puteana*, 59.3∼108.4 MYA between *L. bicolor* and *C. cinerea*, and 109.9∼176.7 MYA between two clades of the four species were used (Floudas et al., 2012). The MCMCtree program in PAML4 was used to estimate the species divergent time with approximate likelihood method.

### Expanded Gene Family and Positively Selected Gene Identification

Gene families were analyzed using CAFÉ v4.2. The most likely ancestral family sizes for each family were estimated, from which we could infer the family in the *G. tsugae* genome was contracted or expanded. In the gene family analysis, 1,000 random samplings for the Monte Carlo resampling procedure were performed and expanded gene family was identified with a cutoff *p*-value of 0.01.

Meanwhile, codon-based methods were used to identify positive selection signal for *G. tsugae* genes. Multiple sequence alignment was used for all coding regions using mafft. Phylogenies of genes were inferred by RAxML with GTRGAMMA model and 200 fast bootstrap replicates. Positive selection in *G. tsugae* genes was then detected using the branch-site model in CodeML program of the PAML software package. Likelihood ratio test *p*-values were computed and adjusted for multiple testing with an FDR threshold of 0.01. The Gene Ontology and KEGG enrichment of expanded gene family and positively selected genes were made by clusterProfiler and DAVID^10^.

### Whole-Genome SNP Variant Identification for *G. tsugae* and *G. lingzhi*

Forty strains used in the genomic analysis of *G. tsugae* and *G. lingzhi* were resequenced using Illumina novaseq platform with an insert size of 350 bp. Resequencing data generated from this study were submitted to GenBank with the project accession of PRJNA751371. Burrows-Wheeler Aligner (BWA) (Li and Durbin, 2009) were used to align the cleaned reads to the *G. tsugae* genome. The mapping rate, sequencing depth, and coverage rate were estimated using SAMtools (Li et al., 2009); single nucleotide polymorphisms (SNPs) and insertion deletions (InDels) were called through GATK (DePristo et al., 2011). The distribution and potential function of SNP in the genome were predicted by ANNOVAR (Wang et al., 2010). According to the SNP location, we have calculated and compared the SNP density for intergenic, upstream, downstream, intron, and exon regions.

A Neighbor-Joining (NJ) tree representing the relationships of individuals was constructed using MEGA (Kumar et al., 2016)^11^ with 100 times of bootstrap resampling. Principal component analysis (PCA) of sample was performed by smartPCA in EIGENSOFT software v5.0 (Price et al., 2006). The genetic clusters inherent in genomes for samples were investigated using K from 2 to 4 with ADMIXTURE v1.3.0 (Alexander et al., 2009).

### Identification and Characterization of Gene-Encoding Terpene Synthases in *G. tsugae*

The annotated protein sequences of *G. tsugae* were used as queries for a hidden Markov model (HMM) search with the databases SwissProt, InterPro (Mitchell et al., 2019), and carbohydrate-active enzyme databases (CAZy) (Lombard et al., 2014) using HMMER. The retrieved sequences were searched against the SMART (Letunic and Bork, 2018) and the National Center for Biotechnology Information (NCBI) Conserved Domain Search Service tool (Lu et al., 2020). ProtParam (He et al., 2019) was used to predict the number of amino acids, theoretical molecular weight (MW), and isoelectric point (PI) of proteins. The exon and intron structures were identified by Gene Structure Display Server by aligning the coding sequence of each gene against the genome sequence. Conserved motifs of the genes were predicted using MEME (Bailey et al., 2006) with default setting; secondary structure and tertiary structure were predicted using Predict Protein (Rost and Liu, 2003) and SWISS-MODEL (Biasini et al., 2014), respectively. MapChart 2.32 (Voorrips, 2002) was used to visualize the contig distributions of gene-encoding terpene synthases in *G. tsugae* based on the starting position of gene and chromosomal length. Sequences were aligned using Cluster X 2.1 (Larkin et al., 2007), and the phylogenetic trees were constructed based on the alignment using the neighbor-joining method (1,000 repeats) with the parameters of the Poisson model, uniform rates among sites, and partial deletion of gaps in MEGA X (Kumar et al., 2018).

The genomic DNA of *G. tsugae* mycelia was extracted as mentioned above, and the total RNA was extracted using EasyPure Plant RNA kit (TransGen, Beijing, China), which was used for gene cloning of terpene synthase-encoding genes. Primers were designed by Primer3Plus (Supplementary Table 2)^12^. PCRs were performed with 2 μl template DNA, 10 μl 2 × Es Taq MasterMix (Dye; CWBIO, Beijing, China), and 0.25 μl of both forward and reverse primer in a 20-μl total volume, and the reaction conditions followed the instructions. RT-RCR products confirmed by agarose gel electrophoresis were then Sanger sequenced by Sangon (Shanghai, China). The sequence alignment was performed using online pairwise sequence alignment tools available at EMBL-EBI^13^.

## Results

### Whole-Genome Sequencing, Assembly, and Annotation of *G. tsugae*

**FIGURE 1:**
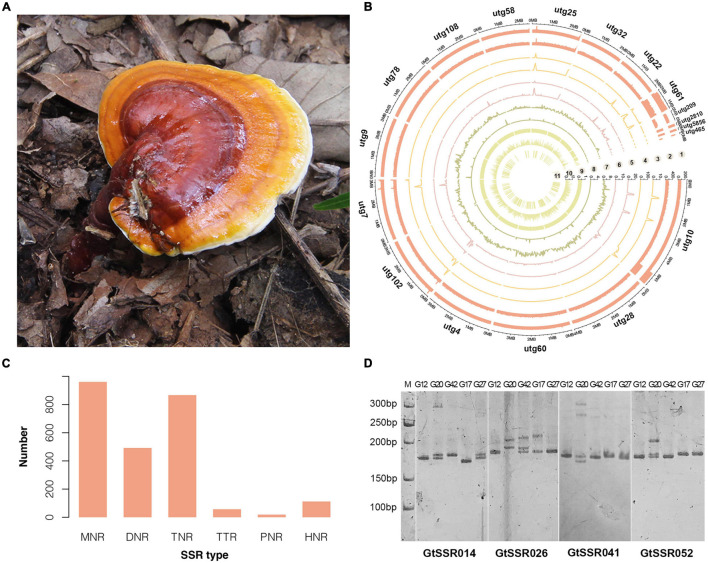
Morphology, genomic landscape and SSR information of *G. tsugae*. **(A)** Fruiting body of wild *G. tsugae*. **(B)** Circos plot of *G. taugse* genome assembly. From outside to inside, track 1, sequence depth for second-generation (Illumina) sequencing; track 2, sequence depth for third-generation (Pacbio) sequencing; track 3–6, homozygous SNPs, heterozygous SNPs, homozygous InDels, and heterozygous InDels detected by second-generation sequencing; track 7, single-copy BUSCOs; track 8, duplicated BUSCOs; track 9, GC content; track 10, gene density; and track 11, location of positive selected genes (PSG). For each track, 50 kb window size was used. **(C)** Distribution to different repeat-type classes. MNR, DNR, TNR, TTP, PNR, and HNR indicate mono-, di-, tri-, tetra-, penta-, and hexa-nucleotide repeats. **(D)** The electrophoretic pattern of partial SSR primers developed for *G. tsugae*.

A high-quality genome of *G. tsugae* (Figure 1A) was assembled from genomic sequencing data of a protoplast-derived monokaryon. We generated 10.48 Gb PacBio long reads with a N50 of 18.45 kb and 5.89 Gb 2 × 150 bp Illumina paired-end short reads, resulting into a ∼219.66 × and ∼123.38 × genomic depth, respectively. The assembly from PacBio long reads generated a 43.26-Mb genome with 18 contigs and a contig N50 of 3.16 Mb (Table 1 and Figure 1B). Due to the advantage of PacBio long reads for the assembly, our newly assembled genome exhibited good continuity on N50 length, which was ∼270 times higher than the N50 length of 11.7 kb for the released *G. tsugae* genome (GCA_003057275.1). By aligning sequencing reads to the genome, 99.55% of Illumina short reads and 96.54% of PacBio long reads were properly mapped. More than 98.5% (1,315 of 1,335) of complete BUSCOs were recovered in the genome assembly, and only 0.75% were missing.

**TABLE 1:** Characteristics of *G. tsugae*, *G. lingzhi*, *G. sinense*, and *G. boninense* genome assemblies.

**Parameter**	***G. tsugae***	***G. lingzhi***	***G. sinense***	***G. boninense***
Source	This study	Chen et al., 2012	Zhu et al., 2015	GCA_002900995.2 in NCBI
Genome size (Mb)	43.26	43.3	48.96	79.19
Number of contigs	18	194	200	495
Contig N50 (Mb)	3.16	0.65	0.68	0.27
Number of scaffolds	–	82	69	–
Scaffold N50 (Mb)	–	1.39	2.26	–
GC content (%)	55.9	55.9	55.6	55.9
Repeat density (%)	10.89	8.15	10.93	13.90
LTR density (%)	5.57	5.42	6.29	8.03
Number of genes	10,946	16,113	15,688	17,553
Average gene length (bp)	1,979	1,556	1,663	1,939
Average number of exons per gene	5.86	4.7	5	5.77
BUSCO (% complete)	98.5	95.5	98.1	89.4

*The statistics for G. lingzhi, G. sinense, and G. boninense were calculated from the available genome data from public database.*

We annotated the genome using a *de novo* and homology-based combined approach. As a result, 10,946 protein-coding genes were identified in the *G. tsugae* genome with an average gene length of 1,978.8 bp (Supplementary Table 3). The average number of exons per gene was 5.9 with an average exon length of 264.4 bp. We also annotated 35 ribosomal RNAs (rRNAs), 241 transfer RNAs (tRNAs), and 20 small nuclear RNAs (snRNAs). In total, 4,709,669 bp repeat elements were identified in our assembly, accounting for 10.89% of the genome. Of them, long-terminal repeat (LTR) retrotransposons were the most abundant class of repetitive DNA with a genome percentage of 5.57%. Among predicted genes in the *G. tsugae* genome, 10,838 (99%) genes were annotated at least in one out of eight databases, namely, KEGG, GO, Nr, Nt, SwissProt, Pfam, and KOG databases.

Then, we identified a total of 2,508 SSR loci among 18 contigs of *G. tsugae* genome. The mononucleotide repeats (MNR) were the most abundant among all six repeat types, which account for 38.32% of total SSRs, followed by trinucleotide repeats (TNR, 34.57%) and dinucleotide repeats (DNR, 19.62%, Figure 1C). To develop SSR markers for *G. tsugae*, we designed 11,645 primer pairs using Primer 3 and selected 100 pairs from them to be examined. The selected SSR loci consist of SSR types from di- to hexa-nucleotide repeats (Supplementary Table 4). Finally, 88 primer pairs were successfully amplified and 59 showed polymorphism among five *G. tsugae* strains (Supplementary Table 4 and Figure 1D). Those SSR markers are valuable resources in further research for *G. tsugae*, for example, in the investigation of QTL mapping toward important agronomic traits.

**FIGURE 2:**
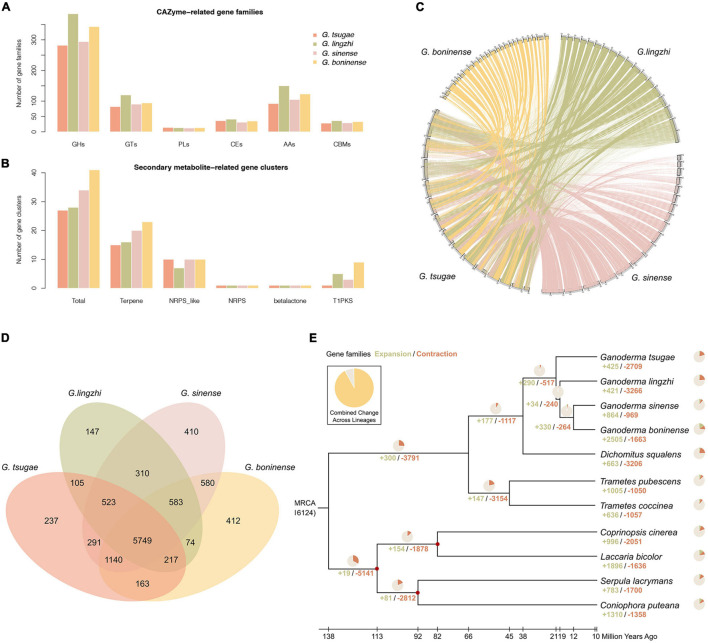
Genome comparison and phylogenetic evolution analysis. **(A)** CAZyme-related gene families in *G. tsugae*, *G. lingzhi*, *G. sinense*, and *G. boninense*. GHs, glycoside hydrolases; GTs, glycosyl transferases; PLs, polysaccharide lyases; CEs, carbohydrate esterases; AAs, auxiliary activities; CBMs, carbohydrate-binding modules. **(B)** Secondary metabolite-related gene clusters in four *Ganoderma* species. NRPS, non-ribosomal peptide synthetase; T1PK, type I polyketide synthases. **(C)** Whole-genome collinearity analysis based on protein-coding genes. **(D)** Venn diagram showing unique and shared gene families. **(E)** Phylogenetic tree and number of expanded and contracted gene families among 11 fungal genomes. Calibrated nodes are indicated by red dots, and the overall timeline is shown below the phylogenetic tree.

Using the CAZy and antiSMASH database, we also annotated genes for CAZymes and secondary metabolic biosynthesis (Figures 2A,B). We found that the annotation of *G. tsugae* was comparable with those of *G. lingzhi*, *G. sinense*, and *G. boninense*, implying the quality of genome assembly and annotation for *G. tsugae*.

With whole-genome protein-coding genes, the conservation synteny of *G. tsugae*, *G. lingzhi*, *G. sinense*, and *G. boninense* were analyzed with collinear of gene arrangement along the genome sequences. In spite of genome fragmentation for those species, especially for public *Ganoderma* genomes, we could still identify clear collinear conservation synteny among those genomes (Figure 2C).

### Gene Family Clustering

Gene family comparison could provide us valuable hints for putative key genes for conifer substrate utilization and medicinal component synthesis for *G. tsugae*. To investigate gene families for *Ganoderma* species, the genomes of *G. tsugae*, *G. lingzhi*, *G. sinense*, and *G. boninense* were used for the genome family analysis. Using whole-genome protein-coding gene sequences, we performed the gene family identification from the sequence clustering analysis (Figure 2D). As a result, we identified 16,124 gene families across those species. Notably, we found that 5,749 gene families with 7,126 genes are shared by all *Ganoderma* species. Interestingly, 237 gene families including 379 genes were exclusively identified in the *G. tsugae* genome. Those genes might relate to the habitat environment adaptation and secondary metabolite synthesis in *G. tsugae*. To reveal putative functions of specific gene families in the *G. tsugae* genome, we performed the functional enrichment for those genes and found that those genes were enriched on amino sugar and nucleotide sugar metabolism, glucagon signaling pathway, degradation of aromatic compounds, and benzoate degradation (Supplementary Table 5).

### Phylogeny and Evolution Among *Ganoderma* Species

To investigate the phylogenetic relationship and divergence time among *Ganoderma* species, we performed the phylogenetic analysis for four *Ganoderma* species, as well as for three other Polyporaceae species (*Trametes coccinea*, *T. pubescenswere*, *Dichomitus aqualens*) and four fossil-recorded fungi species (*Coprinopsis cinerea*, *Coniophora puteana*, *Laccaria bicolor*, and *Serpula lacrymans*; Figure 2E). We performed the gene family clustering for those species and identified 1,240 single-copy orthologs, which was used for the phylogenetic tree construction and divergence time estimation. As expected, *Ganoderma* species were group together. Using the fossil record for *C. cinerea*, *C. puteana*, *L. bicolor*, and *S. lacrymans* as recalibration points, we estimated the divergence time among species in the phylogenetic tree and found that *G. tsugae* speciated from their common ancestor of *G. lingzhi*, *G. sinense*, and *G. boninense* around 21 million years ago, and the genus *Ganoderma* diverged with the genus *Dichomitus* about 38 million years ago.

### Expanded Gene Family and Positively Selected Gene Identification

Using whole-genome protein-coding gene and clustered gene families for all species, we have identified 16 expanded gene families with 151 genes for *G. tsugae*. The functional enrichment analysis showed that many expanded gene families were related to fatty acid metabolism, fanconi anemia pathway, and homologous recombination (Supplementary Table 6). Meanwhile, using gene sequence alignment and statistical analysis, positively selected genes (PSG) were identified in the *G. tsugae* genome. In this work, 184 PSGs for *G. tsugae* were identified with significant positive selection signals (Figure 1B). Interestingly, many PSGs were enriched on mismatch repair, fanconi anemia pathway, base excision repair, and other pathways for DNA repair (Supplementary Table 7). Notably, we also identified several PSGs involved in the important biochemistry pathways, such as glycerol kinase (*glpk*) in the plant-pathogen interaction pathways and Amidase (*amie*) in the aminobenzoate degradation. Those expanded gene families and PSGs might play important roles in physiology and habitat adaptation for *G. tsugae*.

### Population Genomic Analysis Among *G. tsugae* and *G. lingzhi*

To develop genome-wide variants, samples in *G. tsugae* and *G. lingzhi* populations were selected for whole-genome resequencing. Finally, we obtained 937.70∼2,605.23 and 1,203.90∼2,225.63 Mb sequencing data for *G. tsugae* and *G. lingzhi*, respectively, resulting into average sequencing depth of 32.57 and 40.12 for two species (Supplementary Table 1). Using our *G. tsugae* genome as the reference, those sequencing data were aligned and genomic variants were identified as the method described in “Materials and Methods” section.

**FIGURE 3:**
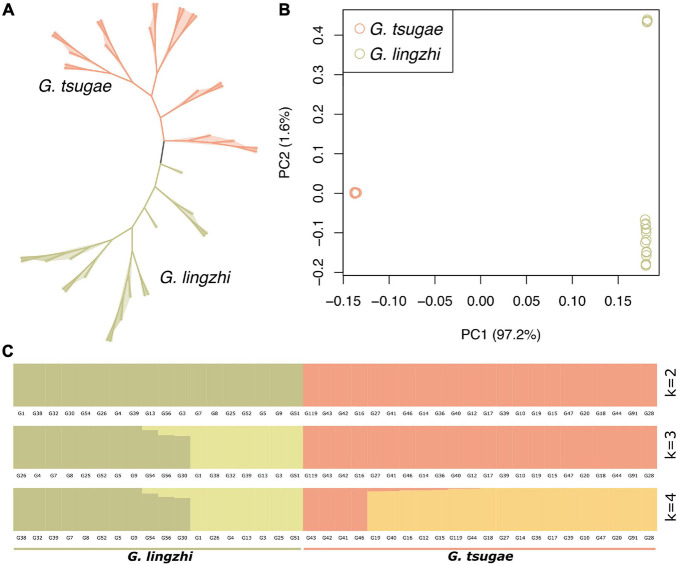
Population analysis using whole-genome resequencing data. **(A)** Phylogenetic tree of 40 strains including 22 *G. tsugae* and 18 *G. lingzhi*. **(B)** Principal component analysis among *G. tsugae* and *G. lingzhi*. **(C)** Population structure analysis showing results when *k* = 2, 3, and 4.

We used whole-genome variants for the clustering analysis for *G. tsugae* and *G. lingzhi*. The variants for samples were filtered so that only loci with sample ratio higher than 80% were retained. As expected, *G. tsugae* and *G. lingzhi* formed two separated clusters in both phylogenetic and PCA analysis (Figures 3A,B). Using whole-genome variants among samples, the genetic structure was analyzed and compared for *G. tsugae* and *G. lingzhi* (Figure 3C). We found that *G. tsugae* and *G. lingzhi* samples exhibited obvious different genetic background. Notably, we cannot separate *G. tsugae* wild and cultivated samples from the genetic structure analysis (Figure 3C), implying resembled genetic backgrounds for wild and cultivated *G. tsugae*.

### Whole-Genome SNP Analysis for *G. tsugae*

**FIGURE 4:**
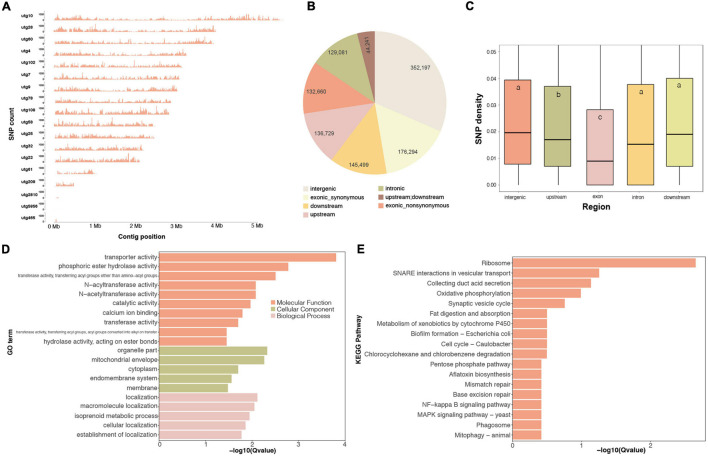
Whole-genome SNP variants on the *G. tsugae* genome. **(A)** SNP density distribution along genome. **(B)** SNP numbers in each genomic regions. **(C)** SNP density for each genomic regions, different letters within the boxes represent significant differences (*p* < 0.05). The GO **(D)** and KEGG **(E)** enrichment for top 1,000 highest SNP density genes.

Whole-genome variants are important genetic resources for functional gene mapping and genetic diversity investigations. In this work, we identified 214,161 high-quality SNP loci for *G. tsugae* from the sequencing data. Those variants were roughly evenly distributed on 18 contigs (Figure 4A). We found that SNP distributions exhibited clear pattern that SNP relatively concentrated at two ends of many contigs, especially for longer contigs. Considering locations of those SNPs, more than 27.7, 12.2, 13.0, 11.6, and 31.5% of loci were residing in exon, upstream, downstream, intron, and intergenic regions, respectively (Figure 4B). According to our result, genomic regions exhibited distinct mutation rate in the *G. tsugae* genomes. The exon region showed the lowest SNP density, which was followed by the upstream region. The SNP density in downstream, intron and intergenic regions were significantly higher than those of exon and upstream regions (Figure 4C and Supplementary Table 8), which could be explained by mutation restraints for negative selection on exon and regulatory regions.

Based on SNP locations and gene coordinates, we could estimate the SNP density for genes. Genes possessing more variants imply abundant genetic diversity among populations. We therefore performed the functional enrichment analysis for top 1,000 genes with the highest SNP density (Figures 4D,E). As a result, GO term of transporter activity, transferase activity, and membrane location were enriched (Figure 4D). Ribosome was also enriched in terms of KEGG pathways, and many transporter and membrane proteins were also identified with high SNP density (Figure 4E), which was consistent with GO enrichment result. The reason for high SNP density on ribosome genes would be twofold. Firstly, ribosome genes have many duplicates in the genome. The mutation on duplicated genes could result into SNP in the variant-calling process. Secondly, previous study has revealed that drastic variations in the rRNA, especially in variable regions that are located on the surface of the ribosome (Xue and Barna, 2012). Abundant biodiversity on transporter genes might relate to the requirement for the transmembrane transportation of diversified small molecules.

### Genome-Wide Identification and Characterization of Genes Encoding Terpene Synthases in *G. tsugae*

**FIGURE 5:**
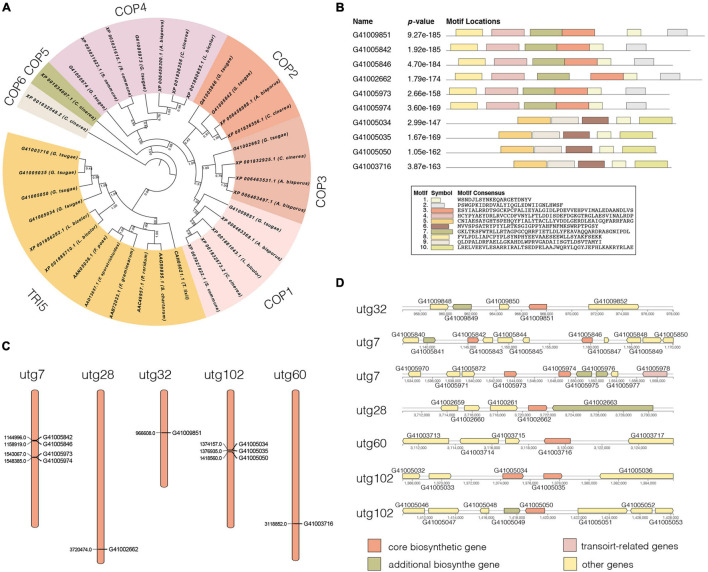
Analysis of terpene synthases genes. **(A)** Phylogenetic tree for terpene synthase genes. Different colors indicate different groups. **(B)** Motif analysis for terpene synthase genes. The length and different colors of boxes denote motif length and different motifs, respectively. **(C)** Distribution of 10 terpene synthase genes on *G. tsugae* contigs. **(D)** Terpene synthase-related gene clusters.

We identified 10 genes encoding terpene synthases in the *G. tsugae* genome. According to the phylogenetic (Figure 5A) and gene structure (Figure 5B) analysis of terpene synthase family proteins, we found that six of 10 genes encoded three kinds of terpene synthases including germacrene A synthase (orthologs with *Coprinopsis cinerea* Cop1 and Cop2), r-muurolene synthase (Cop3), and r-cadinene synthase (Cop4), and other four genes encoded trichodiene synthase (TRI5). Six terpene synthases all contained Terpene_syn_C-2 domain, and four trichodiene synthase contained Isoprenoid_Biosyn_C1 domain. The gene lengths varied from 1,178 to 1,478 bp with 3–6 exons, and the coding sequences (CDS) of the genes ranged from 969 to 1,191 bp (Supplementary Table 9). All the genes were predicted to have α-helix (50.53–61.30%), β-fold (2.90–9.94%), and random coil (34.98–42.78%) structures. The 10 genes were distributed across five contigs of the *G. tsugae* genome, where utg7 and utg102 each had four terpene synthases and three trichodiene synthase genes, respectively (Figure 5C). We found that those 10 genes were from seven gene clusters (Figure 5D). These putative terpene synthase genes provide us a potentially diverse terpenoid metabolism in *G. tsugae*. We also detected SNP variants in the coding and regulatory region of terpene synthase genes (Table 2). The SNP in functional genes, especially for exonic non-synonymous mutations, could be used to develop potential molecular markers for key functional gene mapping and genetic improvement of *G. tsugae* for terpene biosynthesis.

**TABLE 2:** The statistics of variants in functional genes encoding terpene synthases.

**Gene**	**Symbol**	**Upstream**	**Exonic**			**Intronic**	**Downstream**
			**Synonymous**	**Non-synonymous**	**Splicing**		
G41005842	cop2	22	38	10	0	22	64
G41002662	cop3	49	21	54	1	4	47
G41005846	cop2	0	0	0	0	0	0
G41009851	cop1	23	4	4	0	0	27
G41005973	cop4	1	2	1	0	1	11
G41005974	cop4	72	16	33	3	17	99
G41005034	TRI5	0	29	22	0	23	0
G41005035	TRI5	0	4	5	0	2	0
G41005050	TRI5	5	9	3	0	12	19
G41003716	TRI5	18	54	21	0	50	20
							

## Discussion

*G. tsugae*, one of the *Ganoderma* species which was used in traditional Chinese medicine for more than 4,000 years, has been widely cultivated for pharmaceutical product in North China (Yu et al., 2012; Hseu et al., 2019). Using long-read sequencing for protoplast-derived monokaryon, we have assembled and annotated a high-quality reference genome for this important *Ganoderma* species. Our evaluation analysis demonstrated the quality of genome on completeness and continuity. Due to the application of long-read assembly, the contig N50 length of the *G. tsugae* was 3.16 Mb, which was longer than that of *G. lingzhi*, *G. sinense*, and *G. boninense*.

We noticed that less genes were predicted in the *G. tsugae* genome, compared with *G. lucidum* and *G. sinense*. One of reasons could be attributed to the continuity of the genome. The fragmented genome could result into truncated gene predictions and overestimated gene numbers, which was confirmed by the fact that the average gene length and average exon number of genes in our genome is higher than those for *G. lucidum* and *G. sinense*. More importantly, although more genes were predicted in previous studies, approximately 30% (Chen et al., 2012) and 15% (Zhu et al., 2015) of predicted genes failed to hit any homologs in public database, while more than 99% of predicted genes in our *G. tsuage* genome could hit homologs by public database searching.

Using the whole-genome information, the phylogenetic of *G. tsugae* and other related species were analyzed. Divergences for fungi species: *Coprinopsis cinerea*, *Coniophora puteana*, *Laccaria bicolor*, and *Serpula lacrymans* were used as calibration points, and the estimated divergence time among those species were consistent with fossil record. However, we noticed that previous study reported divergence time for *G. lingzhi*–*G. sinense* and *Ganoderma*–*Dichomitus* as about 38 and 62 million years ago (Tian et al., 2021), respectively, which were almost twice as the divergence time in our study. Our result was consistent with the genome study of *G. sinense* (Zhu et al., 2015) that the divergence time for *G. lingzhi*–*G. sinense* and *Ganoderma*–*Dichomitus* were about 18 and 38 million years ago, respectively. We found that *G. tsugae* diverged from their common ancestor of *G. lingzhi*, *G. sinense*, and *G. boninense* around 21 million years ago. Given the fact that *G. lingzhi*, *G. sinense*, and *G. boninense* mainly distribute in south China while *G. tsugae* in north China, we speculate the Himalaya Movement occurred on Eurasian continent about 20 million years ago, and the rise of the Himalaya ranges during the period dramatically change the paleoclimate in south and north China and, thus, might contribute to the divergence of the south and north groups of *Ganoderma* species. However, how tectonic movement and paleoclimate influence the *Ganoderma* evolution still needs more genomic data and further investigations.

Comparing with other *Ganoderma* species, wild *G. tsugae* could grow on coniferous wood. Coniferous wood contains pine oil including terpene and aromatic compounds with antifungal activity (Yang et al., 2008; Noreikaitė et al., 2017); however, the molecular mechanism of the coniferous wood utilization for *G. tsugae* is still obscure. The genome analysis of *G. tsugae* provided useful hints to address this question.

Firstly, coniferous wood likely pose challenges to DNA instability for *G. tsugae*, since ketones, phenols, and other antifungal compounds might induce DNA cross-linking and breakage for pathogens (Lhiaubet et al., 2001). In the *G. tsugae* genome, genes related to fanconi anemia pathway, homologous recombination, and mismatch repair were expanded and positively selected, such as bloom syndrome protein (*blm*), DNA mismatch repair protein (*mlh1*), replication factor C subunit 1 (*rfc1*), DNA polymerase delta subunit 1 (*pold1*), fanconi-associated nuclease 1 (*fan1*), and DNA excision repair protein (*ercc1*). Given their biological functions in DNA interstrand cross-link repair and replications, those expanded and positively selected genes might contribute the DNA stability for *G. tsugae* during coniferous wood utilization.

Secondly, we also found that many PSGs and genes in *G. tsugae*-specific gene families were also likely involved in the alleviation of deleterious influence for *G. tsugae* growth with the coniferous wood. For example, glycerol kinase (*glpk*), a central gene in gluconeogenesis and plant-pathogen interaction (Gottlieb et al., 2014), is positively selected in the *G. tsugae* genome. Glpk is a rate-limiting enzyme that utilizes ATP to catalyze the phosphorylation of glycerol to generate glycerol–3-phosphate (G3P) for glucose production. Therefore, *glpk* might contribute the efficient fat acid-rich coniferous wood digestion. More importantly, G3P is an important molecule signal in plant-pathogen interaction and could prompt salicylic acid level in host plant (Mandal et al., 2011). In one *G. tsugae-*specific gene family, we identified salicylate hydroxylase (*salh*), which could catalyze the hydroxylation and decarboxylation of salicylate to generate catechol. Previous studies showed that *salh* could improve the fungal infectivity and *salh* expression in *Fusarium graminearum* could aggravate Fusarium head blight disease severity for wheat (Makandar et al., 2012). We therefore speculated that *salh* might contribute to the resistance of salicylic acid accumulation for *G. tsugae*. In addition, we also identified many PSGs and genes in *G. tsugae*-specific gene family involved in the degradation of antifungal secondary metabolites. For example, *amie* (Amidase), an important hydrolytic enzyme in detoxification metabolism and aminobenzoate degradation, exhibited significant positive selection signal in *G. tsugae*. Previous study reported that *amie* contributed to the cyflumetofen resistance in *Tetranychus cinnabarinus* (Liu et al., 2020). In addition, *cyp53a1*, *cyp102a*, and *phea* (phenol 2-monooxygenase), identified in *G. tsugae*-specific gene families, were involved in degradation of benzoate, parathion, and phenol (Kirchner et al., 2003; Durairaj et al., 2016). Those expanded and positively selected genes provided valuable clues for the following investigation and validation for key genes contributing to the molecular mechanism of habitat environmental adaptation and coniferous wood utilization for *G. tsugae*.

## Data Availability Statement

The datasets presented in this study can be found in online repositories. The names of the repository/repositories and accession number(s) can be found below: https://www.ncbi.nlm.nih.gov/, PRJNA733861 and https://www.ncbi.nlm.nih.gov/, PRJNA751371.

## Author Contributions

YF designed the research. NJ and SH collected samples and performed the experiments. BP performed genome assembly. SX, ZL, and XY performed the genome analysis. NJ and SX wrote the manuscript, which was reviewed by all authors.

## Conflict of Interest

ZL was employed by ShouXianGu Botanical Drug Co., Ltd. The remaining authors declare that the research was conducted in the absence of any commercial or financial relationships that could be construed as a potential conflict of interest.

## Publisher’s Note

All claims expressed in this article are solely those of the authors and do not necessarily represent those of their affiliated organizations, or those of the publisher, the editors and the reviewers. Any product that may be evaluated in this article, or claim that may be made by its manufacturer, is not guaranteed or endorsed by the publisher.

## Footnotes

1
https://github.com/ruanjue/smartdenovo

2
http://www.repeatmasker.org/RepeatModeler.html

3
http://www.repeatmasker.org/

4
http://tandem.bu.edu/trf/trf.html

5
https://webblast.ipk-gatersleben.de/misa/

6
http://www.kegg.jp/

7
https://www.ncbi.nlm.nih.gov/genome/15719?genome_assembly_id=385534

8
https://fungismash.secondarymetabolites.org/

9
https://www.ncbi.nlm.nih.gov/genome/50735

10
https://david.ncifcrf.gov/

11
http://treesoft.sourceforge.net/treebest.shtml

12
https://primer3plus.com

13
https://www.ebi.ac.uk/Tools/psa/

## Supplementary Material

Supplementary Figure 1 Electrophoretogram of gene cloning and RT-PCR verification of genes encoding terpene synthases. Electrophoretogram of gene cloning **(A)** and RT-PCR detection **(B)**; alignment between the assembly sequence of G41005842 **(C)**, G41005846 **(D)**, G41005974 **(E)**, G41005034 **(F)**, G41005035 **(G)**, G41005050 **(H)** and corresponding RT-PCR products.

Supplementary Table 1 Whole-genome resequencing data for *G. tsugae* and *G. lingzhi* in population genetic analysis.

Supplementary Table 2 Information about primer pairs used in terpene synthases encoding gene cloning.

Supplementary Table 3 Sequence information for functional genes from the *G. tsugae* genome. Note that the first, second, third, fourth, and fifth columns represent gene name, contig id, begin site, end site, FDR for positive selection test, and sequence, respectively.

Supplementary Table 4 Information about 100 primer pairs and corresponding SSR loci.

Supplementary Table 5 KEGG pathway enrichment for genes in the *G. tsugae*-specific gene families.

Supplementary Table 6 KEGG pathway enrichment for genes in the *G. tsugae*-expanded gene families.

Supplementary Table 7 KEGG pathway enrichment for positively selected genes for *G. tsugae*.

Supplementary Table 8 Statistical significance for SNP density between different genomic regions.

Supplementary Table 9 Gene sequence analysis of 10 genes encoding terpene synthases.
