# Supplementary material for: Genome of Ganoderma Species Provides Insights Into the Evolution, Conifers Substrate Utilization, and Terpene Synthesis for Ganoderma tsugae
Source: Front Microbiol. 2021 Sep 16;12:724451. doi: 10.3389/fmicb.2021.724451 (PMC8481371; doi:10.3389/fmicb.2021.724451)
Supplement: Supplementary Figure 1 — Electrophoretogram of gene cloning and RT-PCR verification of genes encoding terpene synthases. Electrophoretogram of gene cloning (A) and RT-PCR detection (B); alignment between the assembly sequence of G41005842 (C), G41005846 (D), G41005974 (E), G41005034 (F), G41005035 (G), G41005050 (H) and corresponding RT-PCR products. [file Data_Sheet_1.docx]

**
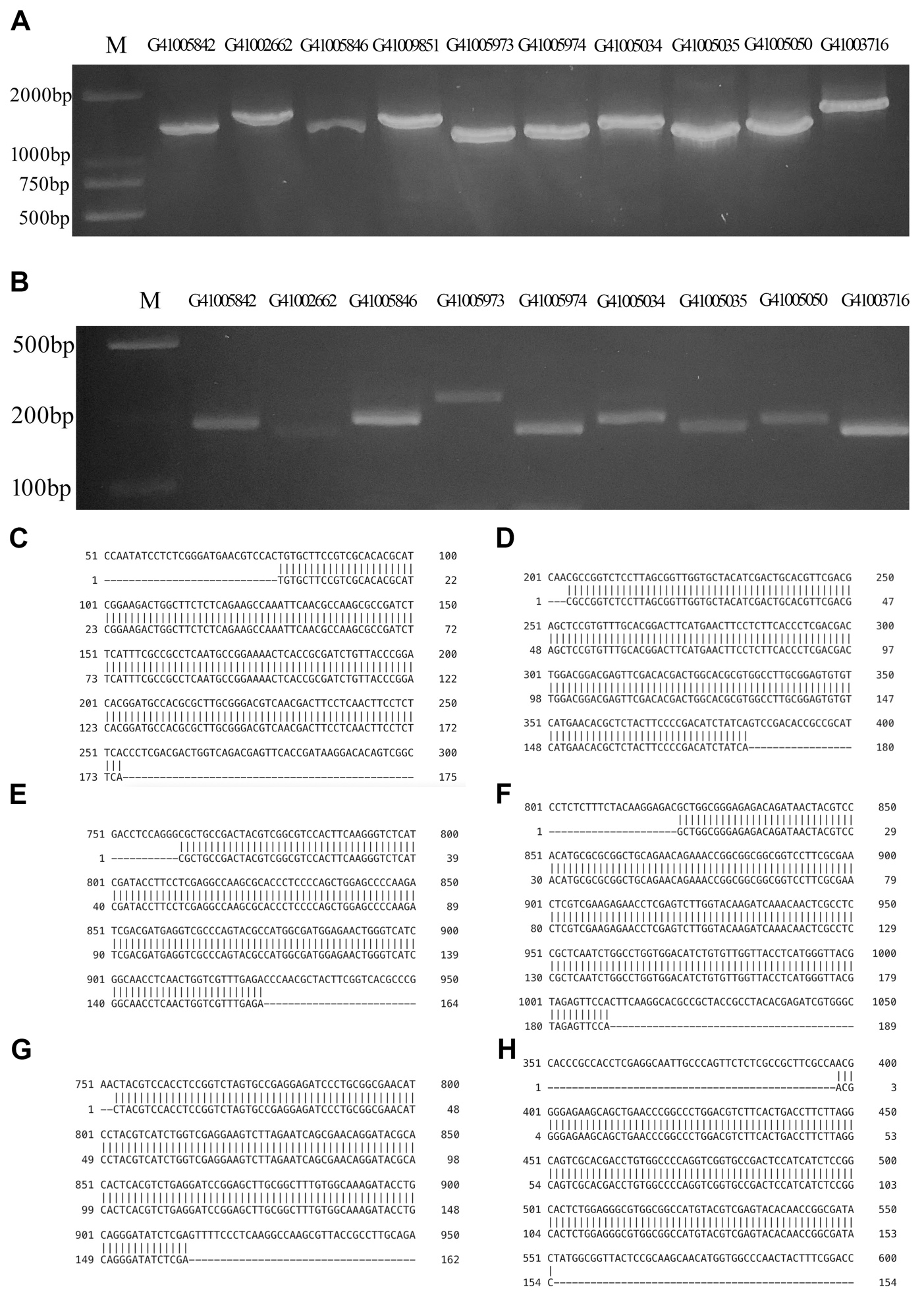
**

**Figure S1. Electrophoretogram of gene cloning and RT-PCR Verification of genes encoding terpene synthases.** Electrophoretogram of gene cloning **(A)** and RT-PCR detection**(B)**; alignment between the assembly sequence (upper) of G41005842**(C)**, G41005846 **(D)**, G41005974**(E)**, G41005034**(F)**, G41005035**(G)**, G41005050**(H)** and corresponding RT-PCR products (lower).

**Table S1. Whole-genome resequencing data for *G. tsugae* and *G. lingzhi* in population genetic analysis (BioProject: PRJNA751371).**

| **Species** | **Sample ID** | **Type** | **Source** | **Collection year** | **Sequencing data (Mb)** | **Mapping ratio (%)** |
| --- | --- | --- | --- | --- | --- | --- |
|  | G10 | Wild | NA | 2013 | 1,468.83 | 93.18 |
|  | G12 | Wild | Guizhou, China | 2014 | 1,042.73 | 93.38 |
|  | G14 | Cultivated | NA | 2014 | 1,435.25 | 95.35 |
|  | G15 | Cultivated | NA | 2014 | 2,605.23 | 94.42 |
|  | G16 | Cultivated | NA | 2014 | 1,091.43 | 93.39 |
|  | G17 | Cultivated | NA | 2014 | 1,073.96 | 93.22 |
|  | G18 | Cultivated | NA | 2014 | 1,413.52 | 95.90 |
|  | G19 | Cultivated | NA | 2014 | 964.98 | 92.90 |
|  | G20 | Cultivated | NA | 2014 | 1,522.75 | 94.15 |
|  | G27 | Cultivated | NA | 2014 | 1,354.35 | 92.67 |
| *G. tsugae* | G28 | Cultivated | NA | 2014 | 1,435.10 | 93.91 |
|  | G36 | Cultivated | NA | 2014 | 1,371.57 | 95.04 |
|  | G37 | Cultivated | NA | 2015 | 1,003.90 | 93.87 |
|  | G40 | Cultivated | NA | 2015 | 1,344.29 | 95.47 |
|  | G41 | Cultivated | NA | 2015 | 1,742.31 | 98.17 |
|  | G42 | Cultivated | NA | 2015 | 1,356.47 | 98.05 |
|  | G43 | Cultivated | NA | 2015 | 1,509.38 | 96.80 |
|  | G44 | Cultivated | Jilin, China | 2015 | 1,434.78 | 93.44 |
|  | G46 | Cultivated | Jilin, China | 2015 | 937.70 | 97.92 |
|  | G47 | Cultivated | NA | 2015 | 1,387.07 | 96.28 |
|  | G91 | Cultivated | Jilin, China | NA | 2,430.06 | 93.48 |
|  | G119 | Wild | Jilin, China | NA | 1,069.35 | 95.15 |
|  | G1 | Cultivated | NA | NA | 2,000.78 | 48.28 |
|  | G3 | Cultivated | NA | NA | 2,196.76 | 47.88 |
|  | G4 | Cultivated | NA | NA | 2,011.35 | 47.73 |
|  | G5 | Cultivated | NA | NA | 2,225.63 | 48.14 |
|  | G7 | Cultivated | Fujian, China | 2012 | 2,112.64 | 47.04 |
|  | G8 | Cultivated | NA | NA | 1,788.99 | 48.11 |
|  | G9 | Cultivated | Jilin, China | 2013 | 1,615.59 | 45.33 |
|  | G13 | Cultivated | NA | 2014 | 1,802.44 | 48.06 |
| *G. lingzhi* | G25 | Cultivated | NA | 2014 | 1,737.34 | 48.42 |
|  | G26 | Cultivated | NA | 2014 | 2,177.56 | 47.70 |
|  | G30 | Cultivated | NA | 2014 | 1,568.11 | 44.18 |
|  | G32 | Cultivated | NA | 2014 | 1,415.70 | 49.92 |
|  | G38 | Cultivated | NA | 2015 | 1,203.90 | 49.66 |
|  | G39 | Cultivated | NA | 2015 | 1,313.44 | 49.67 |
|  | G51 | Cultivated | Shandong, China | 2017 | 1,261.80 | 48.95 |
|  | G52 | Cultivated | Shandong, China | 2017 | 1,501.28 | 47.03 |
|  | G54 | Cultivated | Shandong, China | 2017 | 1,602.76 | 47.66 |
|  | G56 | Cultivated | Shandong, China | 2017 | 1,734.51 | 49.42 |

**Table S2. Information about primer pairs used in terpene synthases encoding gene cloning.**

| **Templete** | **Gene** | **Primer Sequence** | **Product Size (bp)** | **Annealing Temperature (°C)** |
| --- | --- | --- | --- | --- |
| Genomic DNA | G410065842 | ATGGAACGCTCTCCTCGATC | 1319 | 60 |
|  |  | CTATTTTGTCCTGGCGTTTGCA |  |  |
|  | G41002662 | ATGGCCATAGCTGCGTCC | 1478 |  |
|  |  | CTATGCGCCGAGGTGTATGC |  |  |
|  | G41005846 | ATGGCCGTAACTGCTGCTTC | 1298 |  |
|  |  | CTAGGCACGGGCGCGCTG |  |  |
|  | G41009851 | ATGGCTGCCGCTTGCTTTAT | 1421 |  |
|  |  | TCACTTGAGAAGCGGCAGAAG |  |  |
|  | G41005973 | ATGTCCGCGACGAACTTCAG | 1178 |  |
|  |  | TTATTGCGTTGACGGGAGCT |  |  |
|  | G41005974 | ATGTCCGCTCCCAACTCTTC | 1196 |  |
|  |  | TTAGTCCTCCTCGTCAGACG |  |  |
|  | G41005034 | ATGCTTGCCTACCTCAACGC | 1398 |  |
|  |  | TTATGACTGGCCCACGATCTC |  |  |
|  | G41005035 | ATGCTAGCTACACTCTCCAC | 1251 |  |
|  |  | TCAAGGCCGATAGCCCAACT |  |  |
|  | G41005050 | ATGGCCCATACTCAGTCTTCG | 1260 |  |
|  |  | TCACATCAATTCAGCCAGTCG |  |  |
|  | G41003716 | ATGCCTTCCTTCTCTCTTTCCC | 1432 |  |
|  |  | TCAAGCATAGAAACCGAGTTCG |  |  |
| cDNA | G410065842 | GATCGTTCCTTCTCCCTGATCTC | 237 | 57 |
|  |  | TGAAGAGGAAGTTGAGGAAGTCG |  |  |
|  | G41002662 | CATTTTCTCCTACAACGTGGAGC | 211 |  |
|  |  | CCCCTGGATGTACAACGTAACAT |  |  |
|  | G41005846 | CAATCAGAGGAGTGGCTTCTCAG | 250 |  |
|  |  | GATAGATGTCGGGGAAGTAGAGC |  |  |
|  | G41005973 | CTTGTTTCCCACACCTACCCATA | 225 |  |
|  |  | GTAGTCCAAGAGTCGCTGTTTGA |  |  |
|  | G41005974 | CACACTACCAACAACGTGATGAC | 223 |  |
|  |  | TCTCAAACGACCAGTTGAGGTTG |  |  |
|  | G41005034 | CCTGAACTAGAGCTTGTGACCAT | 246 |  |
|  |  | GGAACTCTACGTAACCCATGAGG |  |  |
|  | G41005035 | GCTAGGCACCAATGACATACTCT | 222 |  |
|  |  | TCGAGATATCCCTGCAGGTATCT |  |  |
|  | G41005050 | CTGTACACAGCTTGCTTCCTCTA | 242 |  |
|  |  | ATAGTATCGCCGGTTGTGTACTC |  |  |
|  | G41003716 | CGACCTGCTGTCATTCTACAAAG | 221 |  |
|  |  | TTACGTGGAACTCAAGATAGCCC |  |  |

**Table S5. KEGG pathway enrichment for genes in the *G. tsugae*-specific gene families.**

| **Pathway Hierarchy** | **KEGG Pathway** | **Gene Number** | **Background number** | **P-Value** | **Q-Value** |
| --- | --- | --- | --- | --- | --- |
| Immune system | Cytosolic DNA-sensing pathway | 9 | 25 | 9.32E-08 | 1.36E-05 |
| Transcription | RNA polymerase | 10 | 38 | 9.43E-07 | 6.88E-05 |
| Nucleotide metabolism | Pyrimidine metabolism | 14 | 99 | 6.18E-05 | 3.01E-03 |
| Biosynthesis of other secondary metabolites | Aflatoxin biosynthesis | 8 | 40 | 8.58E-05 | 3.13E-03 |
| Cellular community - prokaryotes | Biofilm formation - Escherichia coli | 2 | 3 | 1.09E-04 | 3.18E-03 |
| Carbohydrate metabolism | Amino sugar and nucleotide sugar metabolism | 12 | 84 | 1.54E-04 | 3.74E-03 |
| Endocrine system | Glucagon signaling pathway | 5 | 20 | 2.51E-04 | 5.24E-03 |
| Lipid metabolism | Steroid hormone biosynthesis | 3 | 8 | 3.10E-04 | 5.66E-03 |
| Overview | Degradation of aromatic compounds | 11 | 80 | 3.72E-04 | 6.03E-03 |
| Biosynthesis of other secondary metabolites | Indole diterpene alkaloid biosynthesis | 2 | 4 | 4.20E-04 | 6.14E-03 |
| Lipid metabolism | Biosynthesis of unsaturated fatty acids | 6 | 33 | 7.83E-04 | 1.04E-02 |
| Lipid metabolism | alpha-Linolenic acid metabolism | 3 | 10 | 8.62E-04 | 1.05E-02 |
| Signal transduction | Hippo signaling pathway -fly | 4 | 17 | 9.37E-04 | 1.05E-02 |
| Signal transduction | Hippo signaling pathway - multiple species | 4 | 18 | 1.25E-03 | 1.30E-02 |
| Xenobiotics biodegradation and metabolism | Benzoate degradation | 6 | 37 | 1.60E-03 | 1.56E-02 |
| Lipid metabolism | Fatty acid elongation | 3 | 12 | 1.88E-03 | 1.72E-02 |
| Signal transduction | cAMP signaling pathway | 5 | 30 | 2.57E-03 | 1.92E-02 |
| Signal transduction | Hippo signaling pathway | 4 | 21 | 2.63E-03 | 1.92E-02 |
| Folding, sorting and degradation | SNARE interactions in vesicular transport | 4 | 21 | 2.63E-03 | 1.92E-02 |
| Sensory system | Phototransduction - fly | 1 | 2 | 2.29E-03 | 1.92E-02 |
| Xenobiotics biodegradation and metabolism | Aminobenzoate degradation | 9 | 84 | 6.46E-03 | 4.49E-02 |

**Table S6. KEGG pathway enrichment for genes in the *G. tsugae*-expanded gene families.**

| Pathway Hierarchy | KEGG Pathway | Gene Number | Background number | P-Value | Q-Value |
| --- | --- | --- | --- | --- | --- |
| Lipid metabolism | Fatty acid biosynthesis | 8 | 8 | 3.54E-16 | 2.83E-15 |
| Overview | Fatty acid metabolism | 8 | 8 | 2.10E-12 | 8.39E-12 |
| Signal transduction | Notch signaling pathway | 4 | 4 | 2.67E-10 | 7.12E-10 |
| Endocrine system | Thyroid hormone signaling pathway | 4 | 4 | 6.30E-07 | 1.26E-06 |
| Replication and repair | Fanconi anemia pathway | 4 | 4 | 1.25E-06 | 1.67E-06 |
| Aging | Longevity regulating pathway - multiple species | 4 | 4 | 1.25E-06 | 1.67E-06 |
| Replication and repair | Homologous recombination | 4 | 4 | 3.90E-06 | 4.46E-06 |
| Cell growth and death | Cell cycle | 4 | 4 | 4.25E-05 | 4.25E-05 |

**Table S7. KEGG pathway enrichment for positively selected genes for *G. tsugae*.**

| **Pathway Hierarchy** | **KEGG Pathway** | **Gene Number** | **Background number** | **P-Value** | **Q-Value** |
| --- | --- | --- | --- | --- | --- |
| Metabolism of cofactors and vitamins | Lipoic acid metabolism | 1 | 3 | 0.00201 | 0.14491 |
| Environmental adaptation | Plant-pathogen interaction | 1 | 4 | 0.00395 | 0.14491 |
| Replication and repair | Mismatch repair | 3 | 29 | 0.006443 | 0.14491 |
| Endocrine system | Renin-angiotensin system | 1 | 5 | 0.006471 | 0.14491 |
| Endocrine system | PPAR signaling pathway | 3 | 32 | 0.009183 | 0.14491 |
| Excretory system | Proximal tubule bicarbonate reclamation | 1 | 6 | 0.00954 | 0.14491 |
| Signal transduction | Hippo signaling pathway - multiple species | 2 | 18 | 0.010764 | 0.14491 |
| Energy metabolism | Nitrogen metabolism | 2 | 19 | 0.01254 | 0.14491 |
| Excretory system | Vasopressin-regulated water reabsorption | 2 | 19 | 0.01254 | 0.14491 |
| Immune system | Toll and Imd signaling pathway | 1 | 8 | 0.017205 | 0.157473 |
| Cell growth and death | Necroptosis | 3 | 39 | 0.018248 | 0.157473 |
| Amino acid metabolism | Phenylalanine, tyrosine and tryptophan biosynthesis | 2 | 22 | 0.018824 | 0.157473 |
| Replication and repair | Fanconi anemia pathway | 3 | 40 | 0.019874 | 0.157473 |
| Glycan biosynthesis and metabolism | N-Glycan biosynthesis | 3 | 43 | 0.025276 | 0.157473 |
| Signal transduction | Calcium signaling pathway | 1 | 10 | 0.02672 | 0.157473 |
| Lipid metabolism | alpha-Linolenic acid metabolism | 1 | 10 | 0.02672 | 0.157473 |
| Digestive system | Pancreatic secretion | 1 | 10 | 0.02672 | 0.157473 |
| Folding, sorting and degradation | Proteasome | 3 | 44 | 0.027255 | 0.157473 |
| Translation | Aminoacyl-tRNA biosynthesis | 3 | 46 | 0.031484 | 0.172332 |
| Replication and repair | Nucleotide excision repair | 3 | 48 | 0.036078 | 0.187566 |
| Metabolism of other amino acids | Selenocompound metabolism | 1 | 12 | 0.037874 | 0.187566 |
| Cell growth and death | Ferroptosis | 1 | 13 | 0.044005 | 0.208025 |

**Table S8. Statistical significance for SNP density between different genomic regions.**

| **Genomic Region** | **P-Value** |
| --- | --- |
| exon - downstream | 0.0000000 |
| intergenic - downstream | 0.8970170 |
| intron - downstream | 0.9999215 |
| upstream - downstream | 0.0000039 |
| intergenic - exon | 0.0000000 |
| intron - exon | 0.0000000 |
| upstream - exon | 0.0000000 |
| intron - intergenic | 0.6942197 |
| upstream - intergenic | 0.0000000 |
| upstream - intron | 0.0000000 |

**Table S9. Gene sequence analysis of 10 genes encoding terpene synthases.**

| **Gene** | **Contig** | **Coordinate** | **Start** | **End** | **Intron**  **number** | **CDS length/ bp** | **Gene family** |
| --- | --- | --- | --- | --- | --- | --- | --- |
| G41005842 | utg 7 | + | 1144996 | 1146311 | 3 | 1125 | Terpene_syn_C_2 |
| G41002662 | utg 28 | - | 3720474 | 3721951 | 4 | 1179 | Terpene_syn_C_2 |
| G41005846 | tug 7 | - | 1158919 | 1160216 | 3 | 1044 | Terpene_syn_C_2 |
| G41009851 | utg 32 | - | 966608 | 968028 | 3 | 1191 | Terpene_syn_C_2 |
| G41005973 | utg 7 | + | 1543067 | 1544244 | 2 | 1029 | Terpene_syn_C_2 |
| G41005974 | utg 7 | + | 1548385 | 1549580 | 2 | 1029 | Terpene_syn_C_2 |
| G41005034 | utg 102 | + | 1374157 | 1375554 | 5 | 1059 | Isoprenoid_Biosyn_C1 superfamily |
| G41005035 | utg 102 | + | 1376935 | 1378185 | 4 | 969 | Isoprenoid_Biosyn_C1 superfamily |
| G41005050 | utg 102 | - | 1418560 | 1419819 | 4 | 972 | Isoprenoid_Biosyn_C1 superfamily |
| G41003716 | utg 60 | - | 3118852 | 3120283 | 5 | 1038 | Isoprenoid_Biosyn_C1 superfamily |
